# Direct Detection and Discrimination of Nucleotide Polymorphisms Using Anthraquinone Labeled DNA Probes

**DOI:** 10.3389/fchem.2020.00381

**Published:** 2020-05-12

**Authors:** Sarah A. Goodchild, Rachel Gao, Daniel P. Shenton, Alastair J. S. McIntosh, Tom Brown, Philip N. Bartlett

**Affiliations:** ^1^Defence Science and Technology Laboratory, Salisbury, United Kingdom; ^2^University of Southampton, Southampton, United Kingdom; ^3^Department for Transport, Great Minster House, London, United Kingdom; ^4^Chemistry Research Laboratory, University of Oxford, Oxford, United Kingdom

**Keywords:** DNA hybridization, redox reporter, anthraquinone, nucleotide polymorphisms, diagnostics, environmental surveillance

## Abstract

A novel electrochemical detection approach using DNA probes labeled with Anthraquinone (AQ) as a reporter moiety has been successfully exploited as a method for the direct detection of DNA targets. This assay uses simple voltammetry techniques (Differential Pulse Voltammetry) to exploit the unique responsiveness of AQ to its chemical environments within oxygenated aqueous buffers, providing a specific detection mechanism as a result of DNA hybridization. This measurement is based on a cathodic shift of the reduction potential of the AQ tag and the concurrent reduction in peak current upon DNA binding. The further utility of this approach for discrimination of closely related DNA targets is demonstrated using DNA strands specific to *B. anthracis* and closely related bacillus species. DNA targets were designed to the *rpo*B gene incorporating nucleotide polymorphisms associated with different bacillus species. This assay was used to demonstrate that the shift in reduction potential is directly related to the homology of the target DNA. The discriminatory mechanism is dependent on the presence of oxygen in the measurement buffer and is strongly linked to the position of the nucleotide polymorphisms; with homology at the terminus carrying the AQ functionalised nucleotide critical to achieving accurate discrimination. This understanding of assay design was used to demonstrate an optimized assay capable of discriminating between *Yersinia pestis* (the causative agent of plague) and closely related species based on the *gro*EL gene. This method is attractive as it can not only detect DNA binding, but can also discriminate between multiple Single Nucleotide Polymorphisms (SNPs) within that DNA without the need for any additional reagents, reporters, or processes such as melting of DNA strands. This indicates that this approach may have great potential to be exploited within novel biosensors for detection and diagnosis of infectious disease in future Point of Care (PoC) devices.

## Introduction

Technologies capable of providing rapid, agile detection and identification of a range of infectious organisms are desirable to support the development of Point of Care (PoC) devices. Detection of nucleic acids from organisms of interest using DNA amplification techniques such as Polymerase Chain Reaction (PCR) is an established and mature approach for identification of bacterial species of interest (Rachwal et al., 2012; Weller et al., 2012, 2016). A wide variety of platforms exist for DNA amplification and detection, and highly integrated technologies (including direct sample to answer) technologies e.g., FilmArray can provide a robust and sensitive capability for screening panels of agents (Poritz et al., 2011). Current PCR technologies have developed to a point where they are well-accepted as a standard tool in clinical laboratories. However, the breadth of information that is generated by current PCR platforms remains limited by the requirement to design specific assays for each agent. Therefore, the capability to screen large panels of organisms requires a large number of assays or the ability to take a multiplexed approach, at the increasing cost of reagents, throughput, and time. Further limitations of PCR platforms lie in the inability to provide detailed species/strain level information in cases where pathogenic and non-pathogenic species of organisms are highly similar in DNA sequence and cannot be discriminated based on PCR amplification alone. This type of information can be provided by rapidly developing sequencing technologies (Dijk et al., 2018; Chiu and Miller, 2019), but the exploitation of these platforms in routine clinical diagnostics is currently challenged by the sample preparation and computational burdens that are challenging in clinical microbiology laboratories (Greninger and Greninger, 2018).

Lower burden options that provide a bridge between these two technology areas include DNA-hybridization based biosensors (Huyghe et al., 2009). Simple DNA hybridization techniques can be coupled to generic PCR amplification of genetic markers conserved across multiple microbial species, such as 16s RNA or *rpo*B, to provide breadth of assay coverage to panels of organisms of interest while including key Nucleotide Polymorphisms that can be mapped back to specific organisms (Wang et al., 2002; Adékambi et al., 2009; Klindworth et al., 2013). This amplification step is then followed by the determination of the homology of the DNA to specific sequences through hybridization to probes within a biosensor to provide identification of the causative pathogen. DNA hybridization can be detected using a range of approaches, including a number of optical techniques such as colorimetric, fluorescent, luminescent, and Raman spectroscopy (Järvinen et al., 2009; Tomlinson et al., 2014; Papadopoulou et al., 2015; Stulz, 2015; Boynton et al., 2017; Nano et al., 2017; Duprey et al., 2018).

Alternatively, electrochemical techniques, which convert a biological recognition event into a digital electrical signal, have also been widely demonstrated for the detection of DNA hybridization. Electrochemical techniques have particular advantages, including the low cost and portability of the associated measurement platforms and can be readily incorporated into devices to produce inexpensive, sensitive, and accurate instruments for diagnostic applications (Blair and Corrigan, 2019; Yáñez-Sedeño et al., 2019). As a consequence, development of novel electrochemically driven approaches for DNA hybridization remains an area of widespread focus for the development of diagnostic devices and *in-situ* environmental detection of clinically important or hazardous microorganisms.

Electrochemically-linked DNA hybridization assays most commonly employ single stranded hybridization probes of DNA or synthetic nucleic acid analogs such as Peptide Nucleic Acid (PNA), which are then immobilized on an electrode or other conductive substrate. The binding of DNA target molecules to these probes can then be measured using a variety of techniques [extensively reviewed in (Gorodetsky et al., 2008; Kang et al., 2009; Hvastkovs and Buttry, 2010; Tosar et al., 2010; Liu et al., 2012; Sontz et al., 2012; Ferapontova, 2018)]. Binding of complementary strands of DNA can act as a barrier to the movement of dissolved electrochemically active species as indirect measurements of DNA binding through the use of techniques such as Electrochemical Impedance Spectroscopy. Alternatively, the formation of a double stranded DNA complex can be reported by exploiting the ability of some metallo or organic intercalators (such as methylene blue, ruthenium, or derivatives of quinone and ferrocene), to bind differentially to single-stranded or double-stranded DNA thus allowing hybridization to be quantified. DNA probes may also be modified by covalent incorporation of electroactive reporters. Careful placement of the electroactive moiety within the DNA structure can then be used to detect not only DNA hybridization, but also the presence of mismatches within that DNA. This could be based on electron transfer through the DNA, or retardation of the flexibility of that DNA probe, impeding transfer of electrons to the electrode surface (Inouye et al., 2005; Pheeney and Barton, 2013; Zwang et al., 2018; Dauphin-ducharme et al., 2019).

DNA hybridization assays using anthraquinone (AQ) as the electroactive reporter are well-described in the literature. AQ is able to act as a selective intercalator into double stranded DNA (Mcknight et al., 2004; Gholivand et al., 2012). As such addition of aqueous soluble derivatives such as anthraquinonemonosulfonic acid (AQMS) has been extensively studied as a reporter for DNA duplex formation demonstrating the ability to discriminate between fully complementary DNA and DNA targets harboring nucleotide polymorphisms (Wong and Gooding, 2006; Batchelor-McAuley et al., 2010; Kowalczyk et al., 2010; Salvatore et al., 2013; Lin et al., 2016). A number of methods for covalently integrating nucleotides modified with AQ groups into DNA probes have also been developed (Yamana et al., 1996; Whittemore et al., 1999; Connors et al., 2003; Narayanan et al., 2004; Orino et al., 2005; Zhao et al., 2007; Abou-Elkhair et al., 2009; Ben Gaied et al., 2009; Balintová et al., 2011). DNA probes carrying these modifications at 3′ and 5′ termini, and within the DNA strand, have been demonstrated to be capable of specific and selective detection of DNA hybridization and discrimination of nucleotide polymorphisms (Yamana et al., 1996, 2001, 2003; Whittemore et al., 1999; Orino et al., 2005; Kumamoto et al., 2008; Balintová et al., 2011; Pheeney and Barton, 2013; Kongpeth et al., 2016).

While AQ is an attractive electrochemical reporter, it has proven less popular than others, such as methylene blue. One of the reasons for this is that the redox potentials of AQ can be influenced by its environment particularly the presence of protons and thus is heavily influenced by pH (Batchelor-McAuley et al., 2010; Guin et al., 2011). In addition, the interaction of quinones with cationic species is well-researched by supramolecular chemists, where host-guest interactions of cations with quinone ionophore groups affecting redox potentials have been exploited in chemical sensing applications (Beer et al., 1994, 1999; Choi et al., 1995). As such, cationic species can have impact on the redox potentials of AQ in aqueous buffers (Li et al., 2011). Furthermore, AQ is able to self-mediate oxygen reduction in oxygenated buffers generating a catalytic reduction peak in electrochemical experiments in the presence of dissolved oxygen (Armitage et al., 1994; Li et al., 2011). Hence the majority of DNA hybridization assays using AQ require use of deoxygenated buffers (Whittemore et al., 1999; Yamana et al., 2001; Wong and Gooding, 2006; Kumamoto et al., 2008; Balintová et al., 2011; Pheeney and Barton, 2013; Salvatore et al., 2013; Lin et al., 2016). This requirement to remove background oxygen limits the easy exploitation of AQ in simple PoC devices. In addition, their discriminatory capabilities are limited to single nucleotide polymorphisms, reducing their exploitation potential for multiple closely related organisms (Kumamoto et al., 2008; Pheeney and Barton, 2013).

When dissolved oxygen is present in the buffer solution analysis is limited to the AQ reduction peak, due to the innate oxygen reduction properties of AQ. Previous studies have demonstrated that the reduction peak is affected by DNA hybridization to non-immobilized PNA probes with a covalently linked AQ reporter or as a intercalator to immobilized DNA probes resulting in reduction of peak current upon DNA hybridization (Kowalczyk et al., 2010; Kongpeth et al., 2016). This effect has not however been studied in surface immobilized DNA probes covalently labeled with AQ and warrants further investigation. In this study we therefore utilize immobilized DNA probes with covalent AQ reporters and demonstrate that this catalytic self-oxidation mechanism can be used as a simple direct reporter of DNA hybridization. We further demonstrate that this innate functionality of AQ is able to facilitate discrimination between multiple nucleotide polymorphisms within the target DNA offering a new simple approach for discriminating multiple species within a single, simple assay.

## Materials and Methods

All chemicals were sourced and used without further purification from Sigma Aldrich (UK) unless otherwise stated. All solutions were prepared using ultrapure Milli-Q water (18 MΩ cm^−1^) according to concentrations stated. All AQ-modified DNA probes were synthesized by ATD Bio, Southampton, UK. Each probe included a 5′ three dithiol immobilization tag and a hexaethylene glycol (HEG) spacer between the thiol groups and the DNA sequence (Mahajan et al., 2008). An anthraquinone moiety was attached to the 3′ terminus, which would be distal to the electrode surface according to previously published methods (Zhao et al., 2007; Mahajan et al., 2009). Target DNA (24 bp) specific to each chosen species was synthesized by Sigma Genosys using standard methods.

### Design of DNA Probes and Target DNA for the *rpo*B Gene of *Bacillus* Species

DNA sequences for the *rpo*B gene were taken from *Bacillus anthracis* Ames (accession number NC_003997.3), *Bacillus cereus* AH187 (NC_011658.1), *B. cereus* B4264 (NC_011725.1), *Bacillus thuringiensis* Al Hakam (NC_008600.1), and *Bacillus weihenstephanensis* KBAB4 (NC_010184.1). Alignments of the *rpo*B genes were performed (Clustal W) to identify areas of variability. A 24 base pair (bp) region of *rpo*B was selected between positions 1,061 and 1,085 (referenced to the *B. anthracis* genome) of the *rpo*B gene. This region was used for the design of probes BA-1, which was fully homologous with *B. anthracis* but contained between 1 and 7 bp mismatch with the other bacillus species (Table 1).

**Table 1:** Sequences of anthraquinone modified DNA probes and target DNA for the *rpo*B gene target of Bacillus species.

**DNA PROBES**	
BA-1	XXX-HEG-5^′^-ACATTAATTACGCGCTCGCCTTCT-3^′^-AQ
BA-2	XXX-HEG-5^′^-ACATTAATTACGCGCTCGCCTTCG-3^′^-AQ
**TARGET DNA**	
*B. anthracis;* BA	5^′^-AGAAGGCGAGCGCGTAATTAATGT-3^′^
*B. cereus* AH187; BC1	5^′^-AGAAGGCGAGCGCGTAATTAA**C**GT-3^′^
*B. cereus* B4264; BC2	5^′^-AGAAGGCGA**A**CG**T**GTAATTAATGT-3^′^
*B. thuringiensis* Al Hakam; BC3	5^′^-**G**GAAGGCGA**A**CGCGTAATTAATGT-3^′^
*B. weihenstephanensis;* BWK	5^′^-AGAAGGCGA**A**CG**ATCT**AT**A**AA**C**GT-3^′^
ALL-BC	5^′^-**G**GAAGGCGA**A**CG**T**GTAATTAA**C**GT-3^′^
3^′^Terminus	5^′^-GGAAGGCGAGCGCGTAATTAATGT-3^′^
NA	5^′^-**T**GAA**AT**C**ACTTAGCT**T**CGCGTC**GT-3^′^

*Underlined bases in target DNA sequences denote mismatches to probe BA-1. XXX indicates three dithiol linker and HEG is hexaethylene glycol*.

### Design of DNA Probes and Target DNA for the *groEL* Gene of *Yersinia* Species

DNA probes (24 bp) for the *gro*EL gene of *Yersinia pestis* were designed to be fully complementary to the genome sequence of *Y. pestis* Nepal516 (accession number gb_CP000305.1). Homologous regions containing nucleotide polymorphisms within these regions were selected from the genomes of the related bacterial species *Y. pseudotuberculosis* (gb_CP000950.1) and *Y. enterocolitica* (emb_AM286415.1) based on previously published work (Papadopoulou et al., 2015). Sequences of probes and target DNA used for each of the *gro*EL (YP1) assays shown in Table 2.

**Table 2:** Sequences of anthraquinone modified DNA probe and target DNA for the *gro*EL gene target of *Y. pestis*.

**DNA PROBE**	
YP1	XXX-HEG-5^′^-GTACCGTCACCCGCAGCATCATTT-3^′^-AQ
**TARGET DNA**	
*Y. pestis*	5^′^-AAATGATGCTGCGGGTGACGGTAC-3^′^
*Y. pseudotuberculosis*	5^′^-**G**AATGA**C**GCTGCGGGTGACGGTAC-3^′^
*Y. enterolytica*	5^′^-**G**AA**C**GA**C**GCTGCGGGTGACGGTAC-3^′^

*Underlined bases in target DNA sequences denote mismatches to probe YP. XXX indicates three dithiol linker and HEG is hexaethylene glycol*.

### Preparation of DNA Functionalised Electrode Surfaces

Two millimeter diameter gold electrodes (CH Instruments) were mechanically polished using a polishing pad (Buehler) and sequential polishing using alumina powder of 0.3 μm then 0.05 μm followed by polishing with a bare polishing pad. In between each polishing stage, electrodes were cleaned by sonication in ultrapure (Milli-Q) water for 10 min. After the mechanical polishing was completed each electrode was further electrochemically cleaned by cycling in 0.5 M H_2_SO_4_ between −0.1 and 1.5 V vs. Saturated Calomel Electrode (SCE) for 60 cycles at 50 mV s^−1^. Immediately after cleaning, electrodes were washed in ultrapure (Milli-Q) water, dried then soaked in probe solutions at a concentration of 1 μM in 10 mM Tris HCl buffer pH 7.5 containing 0.5 M NaCl for 16 h. The electrode surfaces were washed in buffer to remove excess DNA, then soaked in 1 mM mercaptohexanol in 10 mM Tris HCl buffer pH 7.5 containing 0.5 M NaCl for a further 2 h to ensure a passivated surface and a good presentation of the DNA probe in an upright position (Lee et al., 2006). Electrodes were washed in buffer (10 mM Tris HCl pH 7.5 containing 0.5 M NaCl) prior to use. For each experiment, three replicate electrodes were used for each target DNA to ensure that results were consistent for each target DNA product.

### Electrochemical Measurements

All electrochemical measurements were performed using an Autolab PGSTAT302N (Metrohm Ltd). Electrochemical measurements were performed using a standard three electrode cell using bulk gold working electrodes of 2 mm diameter, a saturated calomel (SCE) reference electrode [both from CHI instruments (IJ Cambria)] with a homemade platinum mesh counter electrode (Goodfellow, 0.25 mm wire with 20 × 20 mm mesh).

### Quantification of DNA Immobilized on Electrode Surfaces

The typical immobilization density achieved using probes BA-1 and BA-2 was calculated using the chronocoulometry method of Steel et al. (1998). Initially *Q*_dl_ for DNA functionalised surfaces in buffer containing no [Ru(NH_3_)_6_]^3+^ was determined using a 500 ms reductive pulse from an initial potential of −0.1 V (equilibrated for 60 s) to −0.4 V in 10 mM Tris HCl buffer pH 7.5. [Ru(NH_3_)_6_]^3+^ was then added to the buffer to a final concentration of 50 μM and allowed to equilibrate to the DNA for 10 min. The charge resulting from the presence of the [Ru(NH_3_)_6_]^3+^ was then determined using the same chronoamperometry cycle and used to solve the integrated Cottrell equation (Equation 1; where *Q* is total charge, *t* is the time, *n* is the number of electrons transferred, *F* is the Faraday constant, *A* is the area of the electrode, *D*_*o*_ is diffusion coefficient and $C_{o}^{*}$ the bulk concentration of [Ru(NH_3_)_6_]^3+^ *Q*_*dl*_ is the double layer charge, and Γ_0_ is the surface coverage of bound [Ru(NH_3_)_6_]^3+^).

(1) $$\begin{array}{r} Q=\frac{{2nFAD}_{o}^{1/2}C_{o}^{*}}{\pi^{1/2}}t^{1/2}+Q_{dl}+{nFA\Gamma }_{0} \end{array}$$

A plot of *Q* relative to time for each electrode in the presence or absence of [Ru(NH_3_)_6_]^3+^ was used to calculate the quantity of cation constrained at the electrode surface. This was used to calculate the quantity of immobilized DNA using Equation 2 where Γ_*DNA*_ is the probe surface density in molecules/cm^2^, m is the number of bases in the probe DNA, *z* is the charge of the redox molecule, and *N*_*A*_ is Avogadro's number.

(2) $$\begin{array}{r} \Gamma_{DNA}=\Gamma_{0}\left(\frac{z}{m}\right)N_{A} \end{array}$$

All buffers were purged for at least 30 min with argon prior to use and all experiments were conducted under a blanket of argon. Electrodes were allowed to equilibrate for 10 min under argon purging after the addition of [Ru(NH_3_)_6_]^3+^.

### Direct Measurement of DNA Hybridisation on Anthraquinone Labeled DNA Probes

Electrodes functionalised with DNA were placed in 10 mM Tris HCl pH 8.0 containing 0.5 M NaCl. This buffer was used for the majority of DNA hybridization experiments with the exception of those to investigate the impact of pH on the electrochemical characteristics of the anthraquinone tag where Tris buffers of pH 9.0 and pH 7.0 were also used. In general, the electrochemical experiments were performed in the presence of oxygen. Where removal of oxygen was required, the measurement buffer was purged with argon for 30 min prior to performing any readings, and experiments were conducted under a blanket of argon.

Initial baseline measurements for each functionalised electrode were taken by Cyclic Voltammetry (CV) and Differential Pulse Voltammetry (DPV). CV experiments with probe BA-1 used a starting applied potential of −0.4 moving to −0.7 V at a scan rate of 50 mV s^−1^ (forward scan) followed by a reverse scan from −0.7 to −0.4 V at the same rate. Four repeats of the forward and reverse scans were performed in each measurement. For probe BA-2 the conditions used for CV were identical to BA-1 with the exception of a slightly more negative starting and finishing potential of −0.5 and −0.75 V respectively. For DPV, a starting applied potential of −0.4 V moving to −0.75 V at a scan rate of 50 mV s^−1^ was used throughout. After initial CV and DPV readings had been taken, target DNA was then added to each electrode at a concentration of 1 μM and incubated for 1 h at room temperature to allow hybridization to occur. Non-complementary DNA was added to additional probes as a negative control. CV and DPV measurements were then repeated to determine the effects of DNA hybridization.

### Data Analysis

DPV scans were baseline subtracted using a polynomial multipoint fitting function (OriginPro 9.1, OriginLab, US). The position of the peak was then identified for each scan. The difference between the peak position before, and after, hybridization of target DNA for each electrode was then calculated. Three replicate data points for each condition and for each target DNA on separate electrodes were combined for all *B. anthracis* assays to produce an average shift for each target DNA strand (*n* = 3). For assays using probe YP1 three replicate experiments each containing four replicate electrode surfaces were included (*n* = 12), 95% confidence intervals were calculated from the replicate data sets to show significant differences (*p* ≤ 0.05).

## Results

### Direct Detection of DNA Hybridisation Using AQ Modified DNA

**Figure 1:**
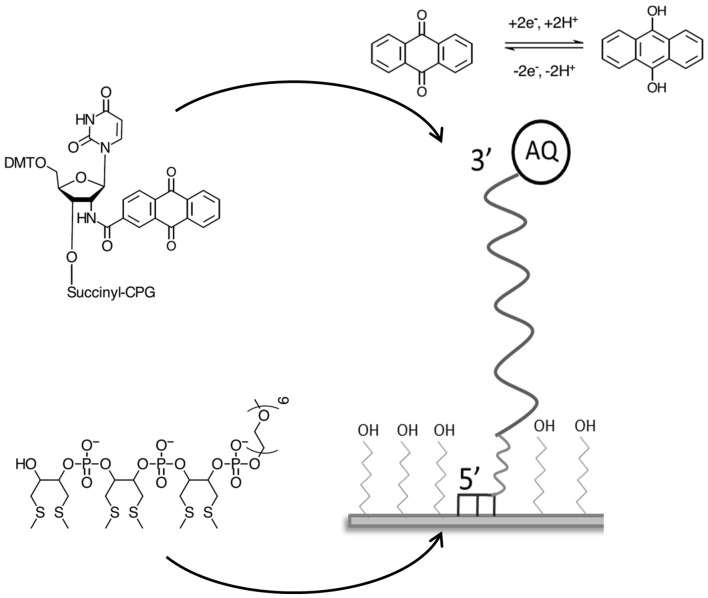
Schematic illustrating the design and format of the AQ DNA hybridization sensor.

DNA probe BA-1 was designed to be specific to a variable region of *B. anthracis rpo*B gene, overlapping SNPs known to exist in other closely related Bacillus species (Table 1). This probe was immobilized on the gold electrode surface via six thiol groups at the 5′ terminus with the 3′ anthraquinone positioned distal to the electrode surface (Figure 1). The density of the probe DNA was measured using chronocoulometry and determined to be 1.17 × 10^12^ (+/– 3.88 × 10^10^) molecules cm^−2^ on average across three identical electrodes. This immobilization density achieved in high salt concentrations is of the level expected for this type of probe based on previous published reports using the same immobilization methods (Mahajan et al., 2008, 2009).

**Figure 2:**
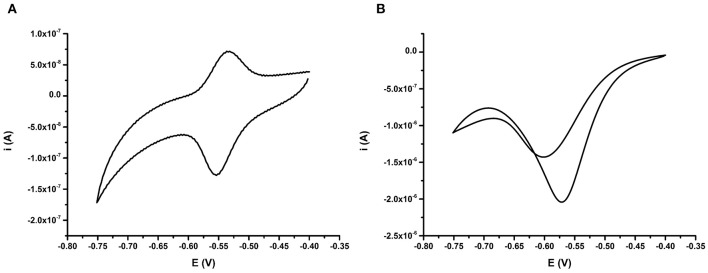
**(A)** Behavior of AQ probe BA-1 deoxygenated (oxygen purged) conditions achieved by purging the electrolyte with argon for 30 min, and **(B)** oxygenated (oxygen present) conditions. Electrochemical scans are representative of *n* = 3 repeat electrodes, and were performed at 50 mV s^−1^ vs. SCE.

The electrochemical characteristics of the anthraquinone functionalized DNA probe BA-1 were subsequently measured using CV. This analysis was undertaken after purging the measurement buffer with argon for 30 min to remove oxygen and also in the presence of ambient concentrations of dissolved oxygen. Clear differences were observed between CVs taken from the same electrode in the presence and absence of oxygen in the solution (Figure 2). In the absence of oxygen, both the oxidation and reduction peaks of the AQ tag, are visible on the CV (Figure 2A). This result provides confirmation that DNA has bound to the electrode surface and that the AQ moiety has remained electrochemically functional. The positions of the oxidation and reduction peaks are also broadly in line with observed potentials from comparable AQ labeled probes being slightly higher than that observed with attachment of AQ to surfaces by self-assembled monolayer (SAM) based immobilization (Nagata et al., 2008) and similar to that observed by other AQ bearing DNA strands using internal and terminal labeling (Yamana et al., 2001; Kumamoto et al., 2008; Pheeney and Barton, 2013).

Conversely when the same electrode was examined in the presence of oxygen (Figure 2B), a change in shape of the CV was clearly observed. In the presence of oxygen a large reduction peak is obvious, but the oxidation peak is missing. This is due to the oxygen reduction capabilities of AQ in oxygenated buffers resulting in a catalytic reduction peak ~10 fold higher than that observed in conditions containing no oxygen. This peak was observed again on the reverse scan suggesting that some structural reorganization of the flexible single stranded DNA probe occurs upon reduction of the AQ tag. Since the AQ tag showed a clear electrochemical response in CV experiments further work was undertaken to understand the impact of the binding of the complementary strand of DNA on the AQ tag.

**Figure 3:**
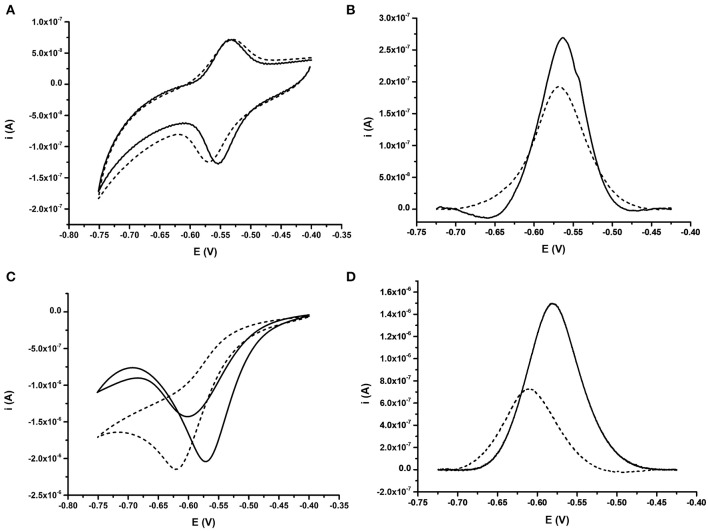
Comparative behavior of probe BA-1 in response to fully complementary DNA under oxygenated and deoxygenated conditions. **(A)** CV and **(B)** DPV for probe under deoxygenated conditions; achieved by purging the electrolyte (10 mM Tris pH 8.0 containing 0.5 M NaCl) with argon for 30 min prior to analysis. **(C)** CV and **(D)** DPV for probe in buffer containing oxygen. In each case the signal from the ssDNA probe modified with AQ is shown as a solid line while the response after hybridization of fully complementary DNA is shown with a dashed line. The response of the same electrode under all conditions is shown. Electrochemical scans were performed at 50 mV s^−1^ vs. SCE.

Fully complementary 24 bp DNA (BA; Table 1) was then allowed to hybridize to DNA probes. Repeat measurements of the CV for the AQ were then taken to understand the impact of the binding event on the electrochemical signature. DPV was also used to interrogate impact of binding specifically on the reduction process of the AQ moiety (Figure 3). The signal from the AQ probe in deoxygenated buffer after hybridization is similar to the pre-hybridization signal. Little effect is observed when comparing CVs measured either before or after DNA hybridization (Figure 3A). When using DPV a slight reduction in the current produced was observed (Figure 3B) and is consistent with published data (Whittemore et al., 1999; Yamana et al., 2001; Kumamoto et al., 2008; Balintová et al., 2011; Pheeney and Barton, 2013). Conversely, with oxygen present in the electrolyte, hybridization of the complementary DNA strand resulted in a large shift in the position of the reduction peak. This shift (53 mV) was clearly observed in both the CV and the DPV (Figures 3C,D, respectively). This was consistently correlated with a drop in the peak current observed (reduction in peak amplitude) observed in the DPV. This is consistent with the signatures observed by Kongpeth et al., when using PNA-AQ functionalised probes in solution (Kongpeth et al., 2016). The CV also showed the appearance of a minor oxidation peak, which was not present prior to hybridization. The combination of these characteristics indicate that the hybridization of the DNA at the probe surface impedes the self-oxidation of AQ and thus slows the electron transfer kinetics of this process which can be observed using these simple electrochemical methods. This exploitation of the native self-oxidation characteristics of AQ offers a new simple methodology. As such, all subsequent measurements using the AQ probes were performed in the presence of dissolved oxygen to target this novel method of detecting DNA/DNA hybridization events. In addition, DPV was used as the simplest measurement technique focusing in on the reduction peak of the AQ probe, exploring effects of DNA hybridization on current output, and peak position.

**Figure 4:**
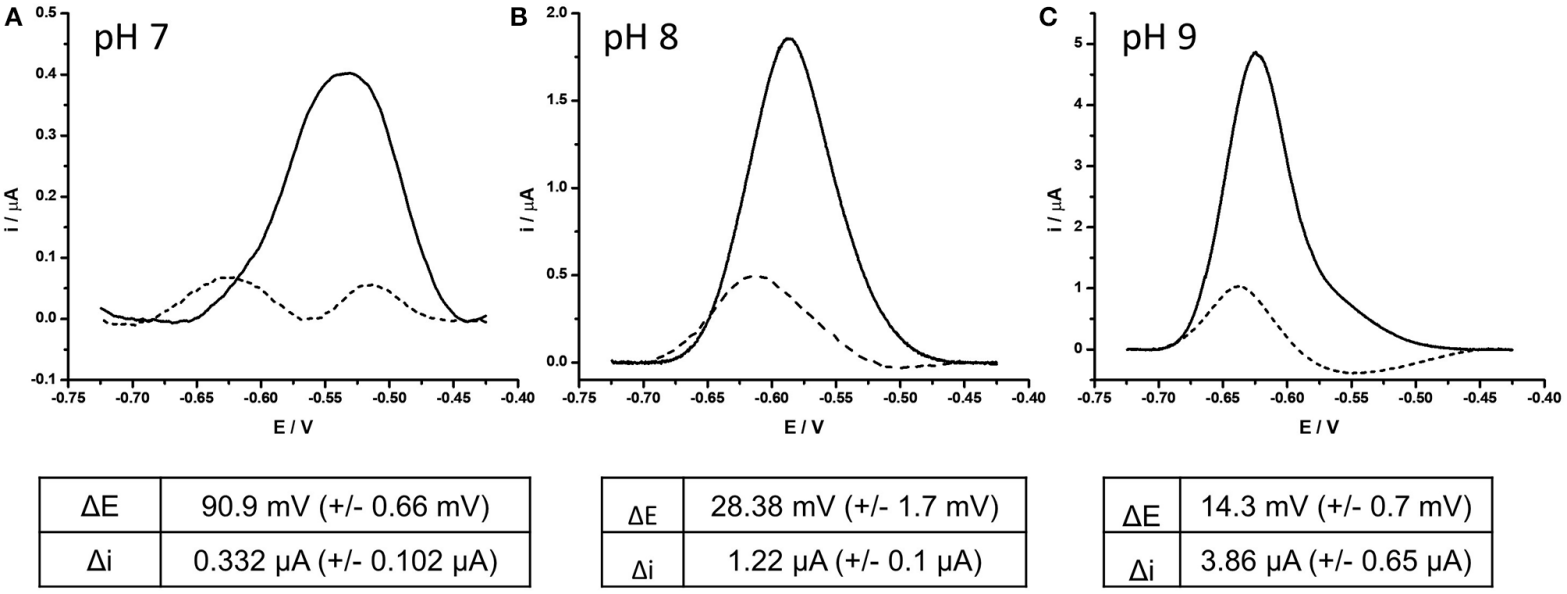
Effect of complementary DNA binding to probe BA-1 at **(A)** pH 7, **(B)** pH 8, **(C)** pH 9. In each case, AQ labeled probe BA-1 was measured as single stranded DNA using DPV (scan −0.4 V to −0.75 V; 50 mV s^−1^ vs. SCE) under oxygenated conditions in measurement buffer 10 mM Tris pH 8, containing 0.5 M NaCl (solid line) prior to hybridization to fully complementary DNA (dashed line). The response of the same electrode under all conditions is shown and is representative of the three electrodes tested under the same conditions.

The involvement of two protons per full electrochemical cycle of the AQ group (Figure 1) means that the pH of the measurement buffer will have a substantial impact on the electrochemistry of the AQ tag. It was therefore considered pertinent to define the impact pH had on the detection of DNA hybridization. DPV analysis of BA-1 was undertaken at pH 7, 8, and 9. Measurements were taken before and after DNA hybridization and the resulting drop in current and reduction potentials compared under each condition (Figure 4). This analysis demonstrated that the presence of excess protons at lower pHs results in a more positive initial redox potential, as well as lowest initial current output. After hybridization of the target DNA, two peaks are observed from the AQ probe at pH 7. The negative shift associated with one of these peaks is extremely large (90.9 mV +/– 0.66 mV) but the presence of an additional peak is a confounding factor in assay output. The reason for two separate peaks has not been further investigated in this work, but was consistently observed at lower pH using AQ labeled probes. We hypothesize this effect to be due to position effects of the DNA relative to the electrode surface at lower pH, which may be more complex in proton rich conditions that offset the natural negative charge of the DNA probe strands and could lead to secondary structures forming within the DNA probe, or less efficient binding of the target DNA to the high density DNA probes at neutral pH (Zhang et al., 2012). At pH 8 this complex effect is removed with a consistent single peak before and after DNA hybridization (28.4 mV +/– 1.7 mV, in this assay system). Current drop was also consistently observed as previously demonstrated.

At pH 9 the initial peak position was the most negative of the three conditions and had the highest current output (x10 relative to that at pH 7). After DNA binding occurred the current drop associated with the binding event was clearly observed, but the shift in position of the reduction peak was minimal (14.3 mV +/– 0.7 mV) reducing the dynamic nature of the signals observed. On the basis of this investigation the decision was taken to perform all future experiments at pH 8, as this condition demonstrated the best balance of current drop, and reduction potential shift.

### Distance Dependence of Binding Event Relative to Current Drop and Reduction Potential Shift

**Figure 5:**
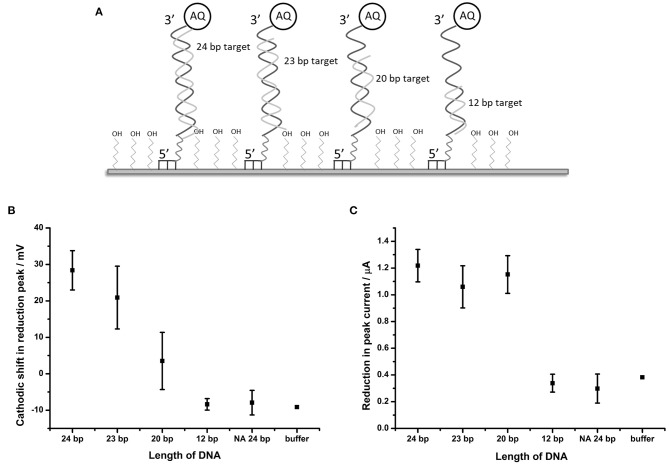
Distance dependent effects of binding complementary DNA to probe BA-1. **(A)** Schematic description of the experimental design showing the position of binding of the shortened DNA strands in relation to the AQ tag. **(B)** Plot showing shift in the potential of the reduction peak and **(C)** plot of the change in peak current in response to hybridization of complementary DNA of different lengths. Points represent an average of the responses shown by three replicate electrodes (error bars indicate 95% confidence limits).

It was considered likely that the effect on the catalytic self-oxidation of the AQ tag would be distance-related i.e. will be reliant on a close interaction of the hybridizing DNA strand to the AQ tag, resulting in occlusion of the AQ tag from oxygenated solvent. To further investigate this hypothesis, DNA targets of 23, 20, and 12 bp were synthesized (these were identical in sequence to BA target DNA described in Table 1, but truncated at the 5′ terminus). Use of these targets would result in complexes where the top of the hybridized strand would be 1, 4, and 12 bp distant from the AQ tag, respectively (Figure 5A).

DPV measurements from BA-1 probe were made in oxygenated buffer before and after hybridization of the target DNA strands. The peak shift and current drop observed after hybridization of each of the probes (*n* = 3) were measured (Figures 5B,C). The maximum shift (28.4 mV) in the reduction peak was observed for a 24 base target which produces a fully double-stranded DNA molecule at the electrode. Smaller changes in the reduction peak were observed for DNA targets of shorter length (19.3 and 5.2 mV for the 23 and 20 bp targets, respectively), which move the proximity of the double stranded DNA away from the AQ tag. A DNA target of 12 bases has no effect on the reduction peak of AQ. A binary response was seen with respect to the peak current (observable as peak height) upon binding DNA targets of different lengths. Targets of 20 bases and longer caused a reduction in peak height of around 1.1 μA, but 12 base targets had the same effect as an entirely non-complementary (i.e., non-binding) 24 bp target or buffer control.

This data suggests that the electron transfer between the electrode and AQ tag is indeed obstructed by the adjacent formation of the DNA double strand. This effect is reduced as the formation of the double strand is withdrawn from the AQ tag. The observations made in this experiment suggest that the electrochemical characteristics of the AQ tag are, as expected, heavily dependent on its environment, specifically in its relationship to hybridized DNA.

### Discrimination Between DNA Targets From Different Bacillus Species Using Probe BA-1

Having demonstrated robust detection of simple DNA hybridization events using an immobilized AQ functionalised probe, attempts were made to define whether the same process could be used to both detect and further discriminate between fully matched DNA sequences and those containing nucleotide polymorphisms. In order to investigate this relationship, an experiment was designed to evaluate the potential application of this methodology to the detection and discrimination of closely related Bacillus species, based on the differences in the conserved gene target *rpo*B.

**Figure 6:**
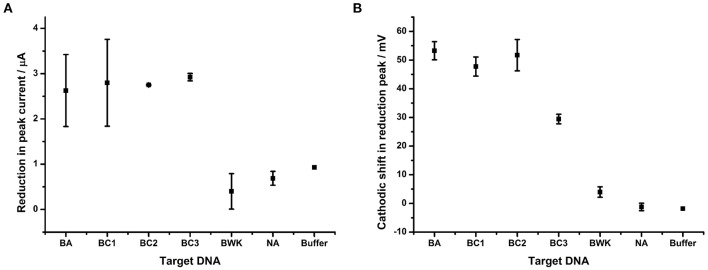
Impact of DNA hybridization on peak current under oxygenated conditions in comparison to reduction peak shift. Probe BA-1 was hybridized to 24 bp DNA strands that were either fully complementary (BA) or contained 1 (BC1), 2 (BC3, BC2), 7 (BWK) nucleotide changes respectively. The response in terms of **(A)** showing reduction in peak current and **(B)** the shift in the potential of the reduction peak, respectively. All measurements were made by DPV (scan −0.4 V to −0.75 V; 50 mV s^−1^) vs. SCE in 10 mM Tris buffer pH 8, 0.5 M NaCl. Points represent an average of the responses shown by three replicate electrodes (error bars are 95% confidence limits from this mean).

The impact of hybridizing each of a series of closely related DNA sequences, given in Table 1, to the probe BA-1 on the reduction of the AQ tag was determined (Figure 6). This experiment demonstrated that peak current appears to be a binary detection signature of DNA binding; i.e., the binding of any target DNA affects the peak current to the same degree irrespective of target homology (Figure 6A). It therefore offers a useful corroborative measurement of overall DNA binding, but does not correlate with homology in this work.

The shift in reduction peak on hybridization of DNA targets does however differ depending on the homology of the target DNA (Figure 6B). DNA corresponding to BC1, from *B. cereus* AH187 (a single nucleotide change) demonstrates a 47.8 mV shift and BC2, from *B. cereus* B4264 (two nucleotide changes) also shows a 51.7 mV cathodic shift in reduction potential. Both of these are indistinguishable from that of *B. anthracis*, BA (53.3 mV, fully complementary). Conversely, hybridization with DNA target from *B. weihenstephanensis* (seven nucleotide changes) produced little effect on the reduction peak with only a 4.0 mV shift. Most interestingly however, hybridization with BC3, *B. thuringiensis*, which like BC2, has two nucleotide mismatches was significantly different from the fully complementary BA target, as the reduction peak is shifted by only 29.5 mV.

**Figure 7:**
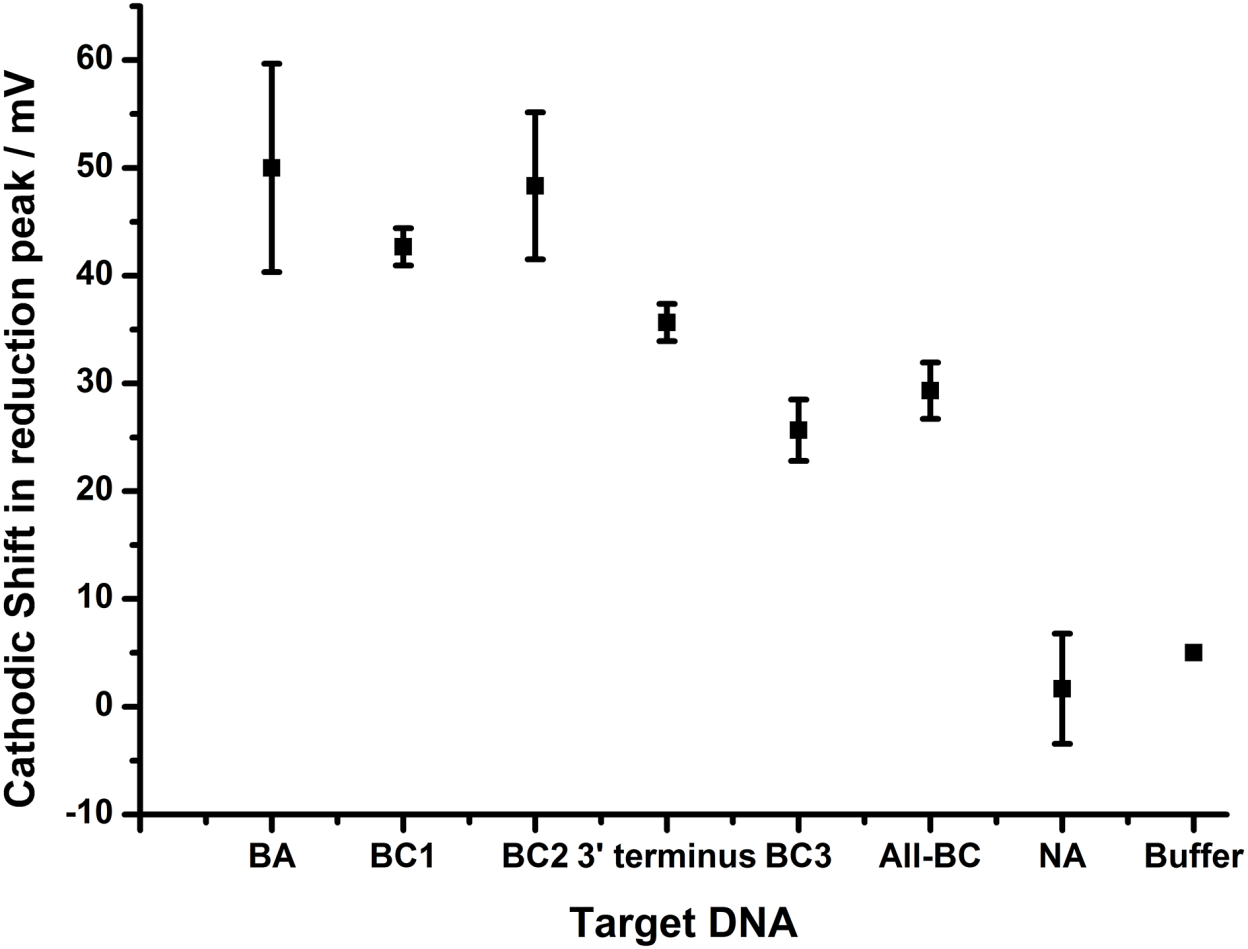
Impact of terminal nucleotide on ability to distinguish base pair changes. Probe BA-1 was hybridized to target DNA that was fully homologous (BA) or contained 1 bp mutation toward the 5′ terminus of probe BA-1 (BC1), 2 bp mutations internal to the BA-1 probe (BC2), single base pair mutation at the 3′ terminus directly associated with the AQ tag (3′terminus), two bp mutations including AQ terminal bp (BC2), a combination of all BC mutations (4 bp mutations All-BC), in comparison to controls NA and buffer only. All measurements were made by DPV (scan −0.4 V to −0.75 V; 50 mV s^−1^) vs. SCE in 10 mM Tris buffer pH 8 containing 0.5 M NaCl. Points represent an average of the responses shown by three replicate electrodes (error bars are 95% confidence limits from this mean).

The difference in the reduction shift between BC2 and BC3 DNA targets, both of which have two nucleotide changes, presents the possibility that the position, or type of DNA mismatch, can affect the extent of the change in the reduction shift. This would be expected as terminal mismatches would be expected to lead to fraying of the DNA strands close to the AQ and thus have greater effect on solvent accessibility than internal mismatches. Further experiments were therefore designed in order to explore this hypothesis and a DNA target termed “3- terminus” containing a single terminal G:T mismatch at the 3′ end of the target DNA was therefore designed and hybridized to probe BA-1 (Figure 7).

The DPV data shows that the shift in the reduction peak for the AQ tag was reduced by 35.7 mV when the terminally mismatched DNA target was hybridized (3′-terminus), in comparison to 50.0 mV for the fully complementary target (BA). When a target containing a terminal mismatch plus one other in the DNA sequence (BC3) was analyzed, a shift in 25.7 mV was observed. This data indicates that the hybridization of DNA target with a single nucleotide change is distinguishable from a fully complementary target if that mismatch is adjacent to the AQ tag. Further nucleotide changes promoting mismatches elsewhere in the double stranded DNA were observed to have an additive effect. This result highlights the potential for this methodology in the robust detection of SNPs when assay design is constrained to include a terminal nucleotide mismatch.

**Figure 8:**
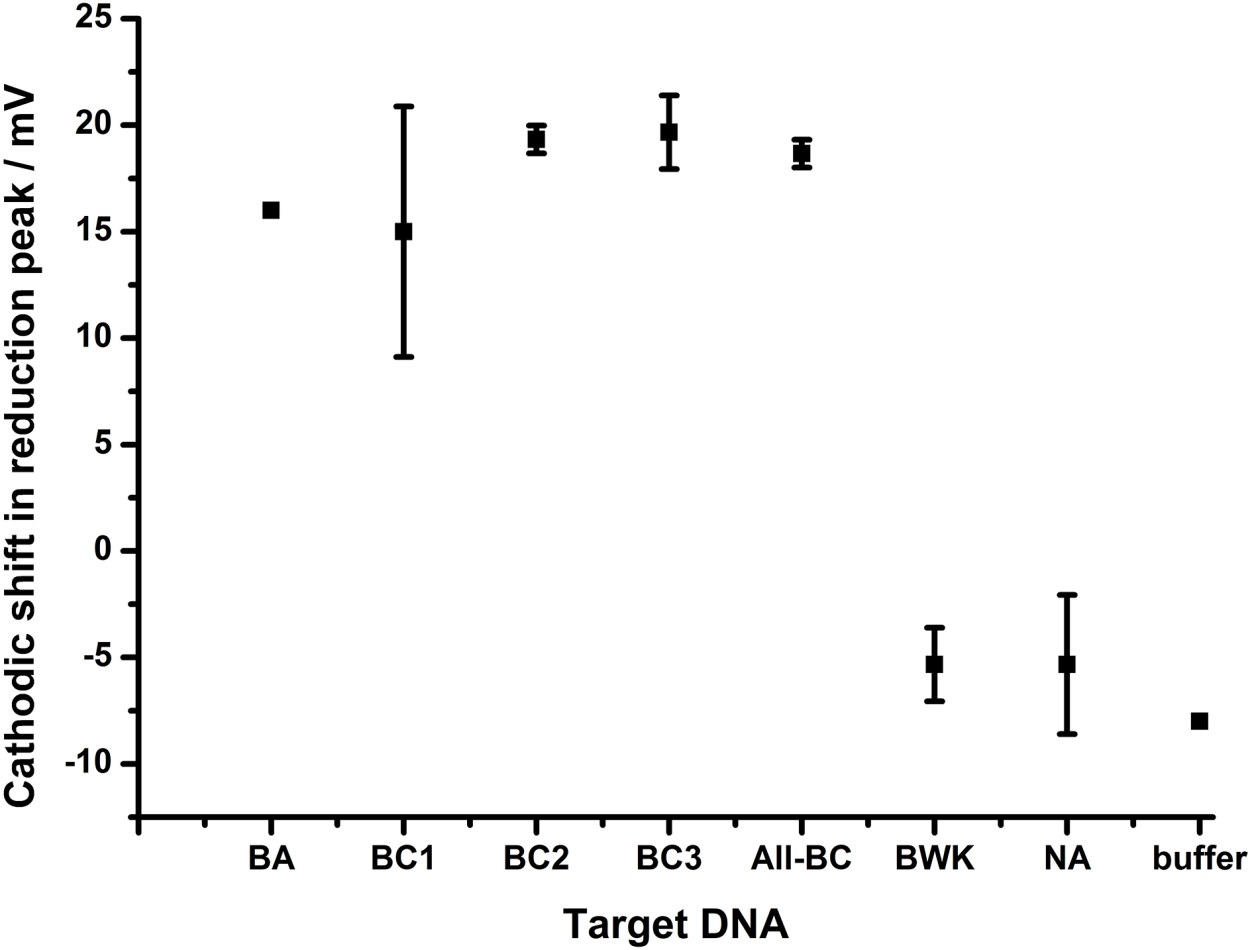
The effect of DNA hybridization to probe BA-2. In each case the 3′ terminal nucleotide carrying the AQ tag is not homologous to any of the target DNA strands. In addition to the terminal mismatch, additional internal mutations were present within target DNA BC1, BC3 (1 bp), BC2 (2 bp), All-BC (3 bp), and BWK (7 bp). All measurements were made by DPV (scan −0.4 V to −0.75 V; 50 mV s^−1^) vs. SCE in 10 mM Tris buffer pH 8 containing 0.5 M NaCl. Points represent an average of the responses shown by three replicate electrodes (error bars are 95% confidence limits from this mean).

The extent of the effect of the terminal mismatch on the electrochemical characteristics of the AQ tag was investigated through the use of an alternative probe, BA-2, which is identical to BA-1 with the exception of the 3′terminal nucleotide which is mismatched to all target DNA used in the previous experiments (Figure 8). This DNA probe deposits on gold electrodes with a similar density to BA-1 at 6.4 × 10^11^ molecules cm^−2^. The data shows that hybridization of all targets produced a similar shift in the reduction peak of the AQ tag (15–20 mV) and that differentiation of the different targets is not observed. This suggests the nature of the terminal mismatch is critical to the mechanism which allows the differentiation of DNA targets observed in previous experiments.

### Assay to Differentiate Between Yersinia Species

Based on the impact of mismatches on the performance of the AQ reporter assay for the *rpo*B target in Bacillus species, a set of design rules were established for assay optimization. Design criteria centered on inclusion of a terminal mismatch between probe and non-homologous targets. Furthermore, additional mismatches were clustered close to the AQ tag where they were expected to have greatest impact on the stability of the DNA complex and thus greatest discriminatory capability. These design rules were used to produce a further optimized assay designed to discriminate between closely related species of the *Yersinia* genus using the *gro*EL housekeeping gene (Table 2).

**Figure 9:**
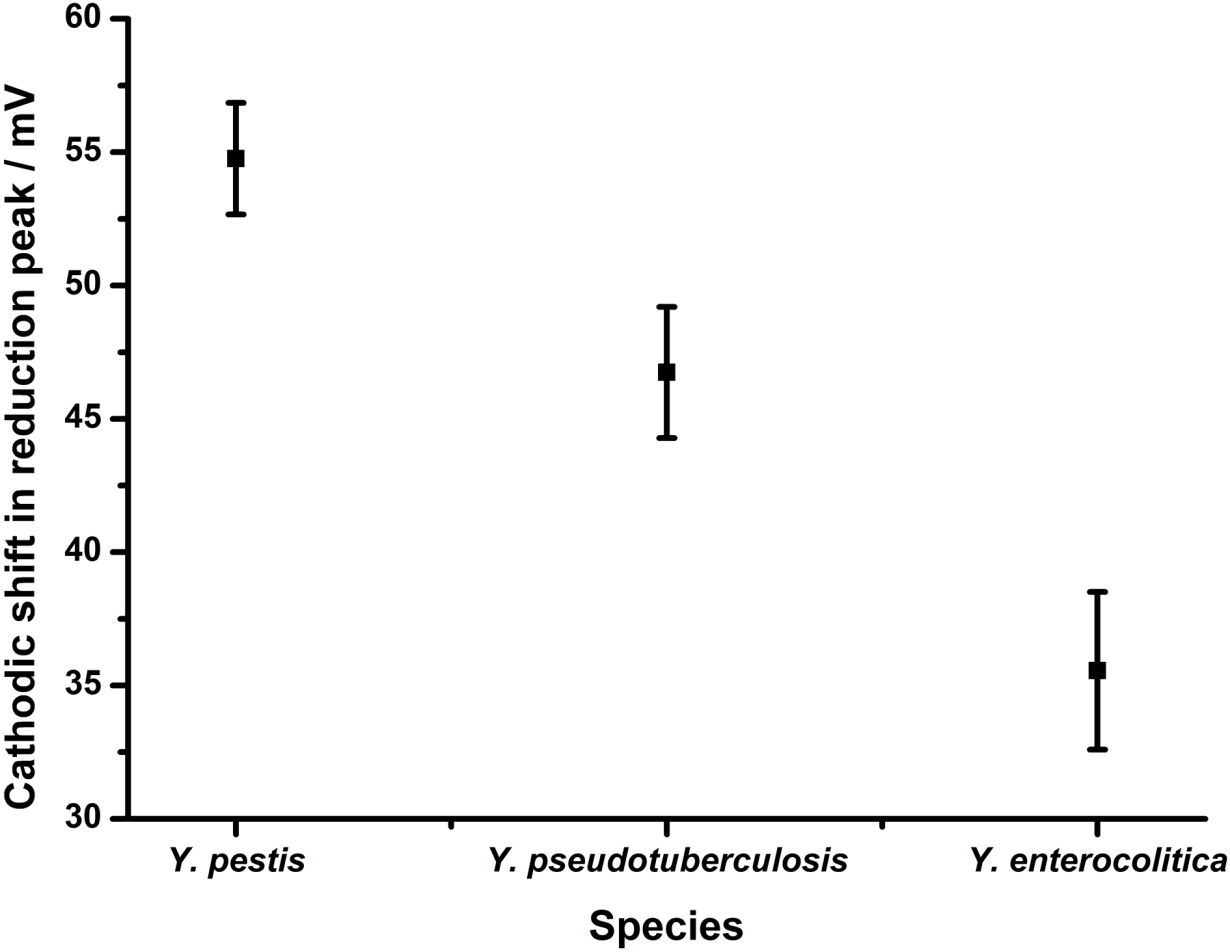
Application of assay design to discriminate between *Yersinia* serovars. Probe YP1 was hybridized to 24 bp strands that were either fully complementary (*Y. pestis*) or containing 2 (*Y. pseudotuberculosis*) or 3 (*Y. enterocolitica*) nucleotide changes respectively. The response in terms of reduction potential shift is shown. All measurements were made by DPV (scan −0.4 V to −0.75 V: 50 mV s^−1^) vs. SCE in 10 mM Tris buffer pH 8, 0.5 M NaCl. Points represent an average of the responses shown by 12 replicate electrodes across three independent experiments (*n* = 4 within each replicate) and error bars are 95% confidence limits.

To evaluate the performance of the *gro*EL assay the YP-1 probe was hybridized to the surface of the gold electrodes as described above and hybridized to target DNA from *Y. pestis* (fully complementary), *Y. pseudotuberculosis* (two nucleotide mismatches), and *Y. enterolytica* (three nucleotide mismatches) (Figure 9). When the fully complementary DNA sequence from *Y. pestis* was applied to the electrode surface, a shift in the reduction potential of 55.8 mV +/– 2 mV was observed. This is consistent with that achieved in the Bacillus *rpo*B assay with fully complementary DNA. The extent of the shift is reduced in line with the reducing complementarity of the DNA targets taken from *Y. pseudotuberculosis* (46.7 mV +/– 2.4 mV) and *Y. enterocolitica* (35.6 mV +/– 2.9 mV). This data suggest that the design rules established using the *B. anthracis* assay could be applied in this assay to discriminate between closely related DNA targets, as differentiation between Yersinia species is achieved depending on the extent of complementarity with the probe sequence. Furthermore, these results are highly reproducible, demonstrating the same response across replicates within the same experiment (*n* = 4) and across three separate experiments, implying this methodology is robust and reliable.

## Discussion

The native oxygen reduction of AQ in aqueous buffers has, to date, led to this tag proving a challenge to exploit as a reporter moiety in DNA hybridization assay requiring purging of electrolyte solutions to gather robust data (Whittemore et al., 1999; Yamana et al., 2001; Wong and Gooding, 2006; Kumamoto et al., 2008; Balintová et al., 2011; Pheeney and Barton, 2013; Salvatore et al., 2013; Lin et al., 2016). Recent reports have however indicated that AQ has the potential to be used as an electrochemical reporter moiety for DNA hybridization assays undertaken in oxygenated electrolytes (Kowalczyk et al., 2010; Kongpeth et al., 2016). The mechanism by which the AQ informs on DNA hybridization in oxygenated buffers, and how this could be applied to both detection and discrimination of nucleotide polymorphisms, has however not been fully investigated in surface immobilized DNA probes.

In this work we have characterized how the formation of DNA duplexes impacts on the electrochemical characteristics of an AQ tag in oxygenated buffers. The AQ tag used in this work is placed as a pendant to the terminus of the DNA probe, using a short linker and therefore is not designed to undergo π stacking into the DNA duplex (Liu and Barton, 2005; Gorodetsky et al., 2007; Pheeney and Barton, 2013). It is therefore unlikely that the signaling mechanism observed is based on electron tunneling through DNA strands. Instead, observations on the effects of DNA targets of different lengths, and the impact of terminal mismatches on AQ reduction potentials, indicates that DNA hybridization affects the innate oxygen reduction of AQ. Thus detection of DNA duplex formation is a combination of solvent occlusion of the AQ tag as a result of the presence of the complementary DNA strand, in addition to likely retardation of electron transfer from the AQ to the surface electrode as a result of the increased rigidity of the DNA duplex over the single stranded probe (Uzawa et al., 2010; Dauphin-ducharme et al., 2019). This mechanism has been demonstrated to be a robust and direct measurement of DNA hybridization.

A detailed study of the effect of terminal DNA mismatches and the additive effects of internal mismatches was undertaken using a model assay designed according to the *rpo*B gene from *B. anthracis*. These observations were used to establish design rules of how to exploit the AQ reporting mechanism to optimal effect. This enabled, bespoke design of an AQ detection assay capable of robustly distinguishing between *Y. pestis, Y. enterocolitica*, and *Y. pseudotuberculosis* using a single AQ labeled DNA probe.

This work has established that electrochemically based DNA probes carrying an AQ tag can be used as a sensor to provide highly discriminatory confirmation of binding events. Data indicates that the redox sensitivity of the AQ tag to its immediate environment can be exploited to provide high resolution information regarding the nature of DNA targets. This ability to discriminate between highly conserved DNA targets can be used to differentiate between closely related bacterial species, providing high confidence output to identify infectious agents of interest with the potential for onward development as a diagnostic technology. Further work will therefore be directed to understanding the performance of this method in terms of sensitivity and specificity against a broader range of targets. In addition, the application of this method to direct detection of DNA from complex sample types to include longer DNA targets produced by PCR assays will be undertaken. This will focus the development of novel technologies exploiting the methodologies identified here.

## Data Availability Statement

The raw data supporting the conclusions of this article will be made available by the authors, without undue reservation, to any qualified researcher.

## Author Contributions

SG and PB devised and designed the study. TB contributed to the DNA design aspects of the study. SG, DS, RG, and AM undertook experimental work. All authors contributed to writing and editing of the manuscript.

## Conflict of Interest

The authors declare that the research was conducted in the absence of any commercial or financial relationships that could be construed as a potential conflict of interest.
